# Synthetic Peptides and Peptidomimetics: From Basic Science to Biomedical Applications—Second Edition

**DOI:** 10.3390/ijms25021083

**Published:** 2024-01-16

**Authors:** Nunzianna Doti, Menotti Ruvo

**Affiliations:** Institute of Biostructures and Bioimaging (IBB), National Research Council (CNR), 80131 Napoli, Italy; menotti.ruvo@cnr.it

Peptides are increasingly emerging as a drug class for a wide range of human diseases due to their intrinsic properties, such as excellent recognition abilities and biocompatibility [1]. To date, the FDA has approved more than 80 peptides, and many others are currently undergoing preclinical and clinical studies [1]. While, in the past, therapeutic peptides were mostly identified from natural sources, more recently they have begun to be selected from rational drug design approaches or from random screening campaigns [2]. For example, antimicrobial peptides (AMPs), which include peptides with antimicrobial, anti-inflammatory, and immunomodulatory activities, are increasingly obtained through multiple approaches that often complement each other [3]. Commonly, the use of natural peptides as pharmaceuticals is significantly limited by several factors, such as low bioavailability, adverse pharmacokinetics, host cell toxicity, and the problems and high costs of large-scale production [1]. Thus, the current challenge is to redesign and chemically synthesize bioactive peptides from natural molecules in order to provide new analogs with reduced toxicity, better pharmacokinetic profiles, and improved bioavailability to implement optimized therapeutic strategies.

This Special Issue, entitled “Synthetic Peptides and Peptidomimetics: From Basic Science to Biomedical Applications—2nd Edition”, provides a collection of five original articles and five reviews describing the design, chemical synthesis, and potential biomedical applications of peptides and peptidomimetics. In one of these, Bellavia et al. (contribution 1) focused on developing a more serum-stable version of the WMR peptide, an AMP derived from the marine peptide Myxinidin, to treat lung infections such as cystic fibrosis, characterized by polymicrobial infections. Then, by testing a series of WVR analogs containing D and/or non-natural amino acids, the authors identified a new peptide with improved serum stability compared to WVR that is capable of inhibiting the formation of both mono- and bi-species biofilms provided by three emerging biofilm microorganisms, namely, C. albicans, S. maltophilia, and A. xylosoxidans. The new molecule worked better than the parent peptide, becoming a good AMP candidate for therapeutic purposes. Dey et al. (contribution 2) reported the design and synthesis of both linear and cyclic short analogs of the marine antimicrobial peptide Turgencin A, isolated from Synoicum turgens and acylated at the N-terminus with octanoic acid (C8), decanoic acid (C10), or dodecanoic acid (C12). The results showed that the highest antimicrobial potency was achieved by introducing C12 or C8 chains into a cyclic analog of Turgencin A, achieving different intrinsic levels of hydrophobicity. The optimized cyclic lipopeptides showed inhibitory concentrations of 4 µg/mL against Staphylococcus aureus, Escherichia coli, and the fungus Rhodothorula sp. Studies on the mechanism of action on bacteria showed the peptide’s ability to rapidly disrupt membrane organization with a consequent bactericidal effect. The hemolytic activity of these molecules against human erythrocytes was low, indicating selective targeting of bacterial cells. Still, in the field of AMPs and their synthetic analogs, Li et al. (contribution 3) provided an overview of the potential healing effects and mechanisms of AMP peptides in three types of lung diseases: acute lung injury, pulmonary fibrosis, and lung cancer.

Peptides or polypeptides with alternate hydrophilic/hydrophobic segments are often used as carriers for drug delivery. Indeed, when they are dispersed in water, they can form micelles, vesicles, hydrogels, and capsules useful for targeted and controlled drug delivery. The design, current synthetic strategies, use of recombinant DNA techniques, polymerization of activated amino acid monomers, and biomedical applications of stimuli-responsive polypeptide-based drug delivery systems have been extensively reviewed by Feng et al. (contribution 4), with an eye toward the technological opportunities in this field. In the same context, Khavinson and coworkers (contribution 5) provided a systematic review of the available data on ultrashort peptides (USPs), consisting of 2–7 amino acid residues, as a drug delivery system involving POT and LAT transporters in various organs and tissues in normal, pathological, and aging conditions.

Another field addressed in this Special Issue is that of peptides and peptidomimetics having immunomodulatory activities by blocking or stimulating the immune response to generate tolerance [4]. Petrović Peroković et al. (contribution 6) focused on the development of mannosylated desmuramyl peptides (ManDMPs) with improved adjuvant activity. They generated mannosylated DMPs with lipophilic substituents attached to the D-isoGln/D-Glu part of the dipeptide pharmacophore via a triazole structure. The introduced modification led to the identification of molecules with adjuvant activities comparable to MDP and better than the benchmark ManDMPTAd. In the same field, Sharapova et al. (contribution 7) described two peptides isolated from the Tag7 protein, acting as novel ligands for the TREM-1 receptor. One of these peptides was able to replicate the function of the entire Tag7 protein, while the other mimicked the receptor-binding region on TREM-1, but was unable to activate signal transduction. The results shown in this work have broadened our understanding of the mechanisms that regulate the innate inflammatory response.

In the field of anticancer drugs, Bojarska et al. (contribution 8) studied the effects of short cyclic peptides containing the sequence Pro-Pro-Phe-Phe on patient-derived melanoma cells. Their report contributed to expanding the knowledge on the preferred bioactivity and reactivity of the cyclo(Pro-Pro-Phe-Phe-) peptide and its analogs obtained through the incorporation of unnatural amino acids. The authors demonstrated that the cyclo(Pro-Pro-Phe-Phe) scaffold can serve as a springboard for obtaining new drugs with effective anticancer activity, as well as new delivery systems of drugs and diagnostics against refractory melanoma.

Numerous in vitro studies on peptides have demonstrated their ability to form amyloid-like structures endowed with special mechanical and spectroscopic properties with enormous implications in the biomedical and technological area [5]. Despite the interest aroused by this phenomenon, the physical basis of its origin is still poorly understood. In this Special Issue, Balasco et al. (contribution 9) present the first comprehensive review of the current literature in which original data on this interesting phenomenon were reported. The review represents a useful source of information for studies aimed at identifying the elusive structural basis of this intriguing phenomenon and at stimulating new applications.

The modulation of protein–protein interactions (PPIs) represents a promising but challenging strategy for finding new therapeutic agents to combat numerous diseases. Peptides, thanks to their ability to mimic the protein regions involved in mutual binding surfaces, represent the ideal class of molecules to address PPI modulation [6]. In this report, Monti et al. (contribution 10) provided a broad overview of the impact of combinatorial chemical approaches on the development of peptide-based compounds to target protein–protein interactions (PPIs) involved in human diseases. After a brief overview of PPIs and the commonly used approaches to identify and characterize them, the authors examined the impact of chemical peptide libraries in medicinal chemistry, focusing in particular on recent results obtained using this methodology. Furthermore, the interesting prospects of chemical combinatorial approaches in the new scenarios opened by the advent of machine learning techniques in structural biology were outlined.
